# The role of serum D-Dimer for the diagnosis of periprosthetic shoulder infection

**DOI:** 10.1007/s00402-022-04385-6

**Published:** 2022-02-19

**Authors:** Doruk Akgün, Faisal Al-Muhtaresh, Alp Paksoy, Lucca Lacheta, Marvin Minkus, Daniel Karczewski, Philipp Moroder

**Affiliations:** Center for Musculoskeletal Surgery, Berlin Institute of Health, Charité – Universitätsmedizin Berlin, Freie Universität Berlin, Humboldt-Universität Zu Berlin, Augustenburger Platz 1, 13353 Berlin, Germany

**Keywords:** Periprosthetic shoulder infection, D-Dimer, Validity, Diagnostics

## Abstract

**Introduction:**

D-Dimer was recently identified as an additional biomarker in the diagnosis of hip and knee periprosthetic joint infection (PJI). Currently, there is only one study in literature dealing with the role of D-Dimer in the diagnosis of shoulder PJI. The purpose of this study was, therefore, to validate the sensitivity and specificity of D-Dimer in detecting shoulder PJI.

**Materials and methods:**

All patients, who underwent septic or aseptic revision shoulder arthroplasty in our institution between November 2018 und March 2021, were analyzed. Our cohort consisted of 30 patients, of that 14 (47%) had a shoulder PJI according the last proposed criteria of the International Consensus Meeting. The diagnostic validity of serum D-Dimer regarding the detection of PJI was analyzed.

**Results:**

The mean D-Dimer level was significantly higher for the patients with shoulder PJI compared to patients with aseptic failure (1.44 ± 1 mg/l vs. 0.76 ± 0.6 mg/l, *p* = 0.025). Coagulase-negative staphylococci were the most commonly isolated pathogens, in 9/14 patients (64%), followed by Cutibacterium acnes in 5/14 patients (36%). According to the ROC analysis, a serum D-Dimer threshold of 0.75 mg/l had a sensitivity of 86% and a specificity of 56% for detection of a shoulder PJI. The area under curve was 0.74. A serum C-reactive protein (CRP) cutoff of 10 mg/l showed a sensitivity of 69% and a specificity of 88%. When both serum D-Dimer and CRP above the thresholds of 0.75 mg/l and 10 mg/l, respectively, were used to identify a PJI the sensitivity and specificity were 57% and 100%, respectively.

**Conclusions:**

Serum D-Dimer showed a good sensitivity but a poor specificity for the diagnosis of shoulder PJI. Combination D-Dimer and CRP led to improvement of the specificity, however, at the cost of sensitivity. Thus, combination of both methods may be used as a confirmatory test in the diagnosis of shoulder PJI but not to rule out infection.

**Level of evidence:**

Diagnostic level II.

## Introduction

Periprosthetic joint infection (PJI) of the shoulder occurs in around 1% of the cases after primary and up to 15% after revision arthroplasty [1, 2] and the absolute number of patients with shoulder PJI is expected to rise with the increasing number of performed shoulder arthroplasties [3]. The ensuing individual as well as social and economic burden is substantial [1]. The timely and correct diagnosis of shoulder PJI holds the key to a successful treatment of these patients with a lasting infection-free survival. Despite the increase of research dealing with the diagnosis of PJI in recent years, shoulder PJI remains a diagnostic challenge due to the ambiguity presented by its unique microbiologic profile with low-virulent microorganisms and stealth-type clinical appearance [4, 5]. Widely used biomarkers in the diagnosis of PJI, such as C-reactive protein (CRP) or erythrocyte sedimentation rate (ESR) have a very limited value in the detection of low-grade infections misdiagnosing more than one third of the patients [6–9]. Therefore, serological tests that can help to diagnose PJI with a higher accuracy are of interest.

Recently, D-Dimer was described as a promising marker for the diagnosis of hip and knee PJI by Shahi et al. [10] and declared as a minor criterium in the consensus definition of knee and hip PJI [11]. However, there is scarce and conflicting evidence supporting its use for the diagnosis of PJI in recent literature, which shows a very limited diagnostic value compared with traditional biomarkers (ESR and CRP) [12–14]. Although the number of studies reporting on the role of D-Dimer in the diagnosis of hip and knee PJI are increasing, currently there is only one study in literature dealing with the role of D-Dimer in the diagnosis of shoulder PJI, showing a limited diagnostic utility with a sensitivity and specificity of 61% and 74%, respectively [15]. The purpose of this study was, therefore, to validate the sensitivity and specificity of D-Dimer in detecting shoulder PJI.

## Materials and methods

### Study design and cohort

This was a cross-sectional study with analysis of collected data of 32 patients, who underwent septic or aseptic revision shoulder arthroplasty in our institution between November 2018 und March 2021. Exclusion criteria were recent history of trauma or previous surgery, as well as history of venous thrombosis, other ongoing infections or coagulation disorder including malignancy or autoimmune diseases. Excluded were two patients with a history of trauma (within 2 weeks) and with a previous surgery within 2 weeks prior to revision surgery, so our cohort consisted of 30 patients. The study protocol was reviewed and approved by the institutional ethics committee (EA4/040/14).

Following data was recorded prospectively for each patient: gender and age, involved joint, clinical symptoms, surgical history of the involved joint, type of arthroplasty, time interval between primary arthroplasty and revision surgery, concurrent antibiotic treatment, as well as radiological assessment.

### Standard diagnostic protocol and definition of shoulder PJI

The standard diagnostic protocol to identify PJI included the following assessments in all patients; Laboratory values including C-reactive protein (CRP), serum D-Dimer, serum leucocyte count as well as results of preoperative aspiration, if performed, including leucocyte count, neutrophil percentage, microbiologic and histopathologic results. Furthermore, radiological and intraoperative evaluation of the component loosening and intraoperative findings, such as cloudy fluid or gross intra-articular purulence were performed and documented.

Preoperative aspiration was performed routinely in every case and was successful in 17 patients (57%). According to the standard PJI protocol at our institution at least 5 periprosthetic tissue cultures from various suspicious surgical sites, at least one specimen for histopathologic analysis and retrieved implants for sonication analysis were obtained in every patient at the time of revision surgery. Specimen for microbiological analysis were collected with a new sterile instrument each time, were placed directly into sterile containers without touching by hand and sent immediately with retrieved implants to our microbiology laboratory for further analysis within 1 h after surgery. The microbiologic specimen as well as sonication fluid cultures are plated onto aerobic and anaerobic sheep blood agar plates and incubated for 14 days. Sonication was performed as previously described [16]. Shoulder PJI was diagnosed according to the last proposed definition criteria of the ICM [17].

### Statistical analysis

Chi-squared and Fisher’s exact tests were used to find significant differences between categorical variables. The two-sample *t* test (for parametric distribution) or Mann–Whitney *U* test (for non-parametric distribution) was used to compare continuous variables between groups. For analysis of the diagnostic utility of serum D-Dimer, patients in the definitive, probable and possible infection groups were combined and defined as infection group and patients in infection unlikely group were defined as non-infection group. Sonication fluid cultures were incorporated into the infection criteria as microbiologic results used for the infection definition. Receivers operating characteristic (ROC) analysis was performed to display sensitivity and specificity of serum D-Dimer level for shoulder PJI. The area under the curve was calculated, and the optimum cutoff point was determined by the maximized Youden`s index. The results were expressed as mean and standard deviation (SD) or as number and percentage. A *p* value < 0.05 was considered significant. SPSS version 20 (SPSS Inc., Chicago, Illinois) was used for the statistical analyses.

## Results

The mean age and standard deviation (SD) of the patients were 69.1 ± 10.4 years and 18 patients were females (60%). Ten patients (33%) had a previous revision surgery, three patients due to a septic reason. The mean interval between the primary arthroplasty and revision surgery were 4.9 ± 6.2 years. The type of arthroplasty at the time of revision surgery was hemiarthroplasty in nine patients, total shoulder arthroplasty in three and reverse shoulder arthroplasty in 18 patients. None of the patients had a history of venous thrombosis or coagulation disorder including malignancy or autoimmune diseases. The reasons for revision surgery included beside infection, loosening of the components, overstuffing, secondary rotator cuff insufficiency and instability.

A total of 14 patients were (47%) were identified as infected, 6 meeting the criteria of definitive infection, 6 of probable infection and 2 of possible infection. The mean D-Dimer level was significantly higher for the patients with shoulder PJI compared to patients with aseptic failure (1.44 ± 1 mg/l vs. 0.76 ± 0.6 mg/l, *p* = 0.025), as well as the mean CRP level (26 ± 30 mg/l vs. 4.2 ± 4 mg/l, *p* = 0.009). A detailed demographic and clinical comparison of both groups is demonstrated in Table 1.

**Table 1:** Patient demographic and clinical characteristics

Variable	Infection group, *n* = 14	Non-infection group, *n* = 16	All patients, *n* = 30	*p* value^a^
Age at revision surgery (year) ∗	72.4 ± 10	66.2 ± 10	69.1 ± 10	0.1
Sex^⧫^				
Male	5 (36)	7 (44)	12 (40)	0.7
Female	9 (62)	9 (56)	18 (60)	
Type of arthroplasty^⧫^				
Hemi	3 (21)	6 (38)	9 (30)	
TSA	3 (21)	0 (0)	3 (10)	
RSA	8 (57)	10 (62)	18 (60)	
Interval between index and revision arthroplasty (year) ∗	6.3 ± 8	3.6 ± 5	4.9 ± 6.2	0.3
CRP at admission (mg/l)∗	26 ± 30	4.2 ± 4	14.3 ± 23	0.009
D-Dimer at admission (mg/l) ∗	1.4 ± 1	0.76 ± 0.6	1.1 ± 0.9	0.025

*TSA* Total shoulder arthroplasty, *RSA* Reverse shoulder arthroplasty, *CRP* C-reactive protein

∗The values are given as the mean and the standard deviation

^⧫^The values are given as the number with the percentage of the group in parentheses

^a^The statistical analysis was only done between infection and non-infection groups

In all but two of infected patients at least one microorganism could have been identified and in 5 patients a polymicrobial infection was evident. Coagulase-negative staphylococci were the most commonly isolated pathogens, in 9/14 patients (64%), followed by Cutibacterium acnes in 5/14 patients (36%), Bacillus cereus in two patients and Staphylococcus aureus and Streptococcus parasanguinus each in one patient. The mean time to revision from the primary arthroplasty was longer for the infection group than non-infection group, however, statistically not significant (6.3 ± 8 years vs. 3.6 ± 5 years, *p* = 0.3). According to the ROC analysis, a serum D-Dimer threshold of 0.75 mg/l had a sensitivity of 86% and a specificity of 56%, a positive predictive value (PPV) of 63%, a negative predictive value (NPV) of 82% and an accuracy of 70% for detection of a shoulder PJI. The area under curve (AUC) was 0.74 and the ROC analysis had a significance level of 0.026. Table 2 shows different thresholds with sensitivity and specificity. A serum CRP cutoff of 10 mg/l as recommended by ICM [17], showed a sensitivity of 69% and a specificity of 88%, a PPV of 82%, a NPV of 79% and an accuracy of 80%. When both serum D-Dimer and CRP above the thresholds of 0.75 mg/l and 10 mg/l, respectively, were used to identify a positive result the sensitivity, specificity, PPV, NPV and accuracy were 57%, 100%, 100%, 73% and 80%, respectively.

**Table 2:** Sensitivities and specificities for different D-Dimer thresholds

D-Dimer threshold (mg/l)	Sensitivity (%)	Specificity (%)
0.24	100	6
0.31	100	19
0.4	100	44
0.66	86	44
0.75	86	56
0.85	71	62
0.9	64	62
1	43	75
1.7	36	94
2.5	14	94
3.1	7	100

## Discussion

Shoulder PJI presents a unique diagnostic challenge because of the low virulence of the most common causative microorganisms. The determination of the infection status in patients with a clinically failed shoulder arthroplasty is a key step in the treatment planning. Therefore, the identification of potential biomarkers for an accurate diagnosis of shoulder PJI is of importance. Recent research focused on the role of D-Dimer in the diagnosis of knee and hip PJI with conflicting results. To our knowledge there is only one study evaluating the role of serum D-Dimer in the diagnosis of shoulder PJI. Our results showed a lower accuracy of D-Dimer compared to CRP in the diagnosis of shoulder PJI, D-Dimer with a higher sensitivity and CRP with a higher specificity. Combination of both methods led to improvement of the specificity, however, at the cost of sensitivity. Thus, combination of both methods may be used as a confirmatory test in the diagnosis of shoulder PJI.

D-Dimer is mostly used as a screening tool for deep vein thrombosis or pulmonary embolism in lower limbs [18]. Recent literature showed an increase of D-Dimer in the setting of systemic inflammation and infection, especially in a joint [19, 20]. Rodelo et al. reported on the prognostic role of D-Dimer in patients with sepsis showing that high level of D-Dimer was associated with an increased 28-day mortality [21]. Shahi et al. first reported on the role of D-Dimer in the diagnosis of hip and knee PJI in a prospective study [10]. They showed a sensitivity and specificity of 89% and 93%, respectively, for a determined optimal threshold value of 0.85 mg/l, which is similar to the value identified in our study by the Youden`s index. Furthermore, they concluded that D-Dimer was also accurate in predicting the presence of infection at the time of reimplantation during a two-stage exchange arthroplasty. In another study Quin et al. identified the D-Dimer as a valuable biomarker in detecting chronic hip and knee PJI with a sensitivity and specificity of 93% and 75%, respectively, with an optimal threshold of 1.170 mg/l. In contrast to our study the authors showed, that a combination of D-Dimer with CRP led to improvement of sensitivity (98%), however, at the cost of specificity (42%). Thus, they recommended use of the negativity of serum D-Dimer and CRP to rule out a PJI [22]. Contrary to these findings, Hong Xu et al. reported a lower sensitivity and specificity of serum D-Dimer (69% and 51%, respectively) compared to CRP, ESR and interleukin-6 with a threshold of 1.02 mg/l [12]. Thus, they stated that D-Dimer has a limited value for diagnosing hip and knee PJI. Similar to these results, Rui li et al. found a lower sensitivity and specificity of plasma D-Dimer (64% and 65%, respectively) in the diagnosis of knee and hip PJI compared to CRP and ESR. They also analyzed plasma fibrinogen, another coagulation-related indicator, and showed a promising performance similar with CRP and ESR. In another study of Pannu et al. serum D-Dimer showed a poor accuracy to discriminate between septic and aseptic cases in the setting of hip and knee arthroplasty revision [14]. Zmistowski et al. were recently able to show a limited diagnostic utility of serum D-Dimer similar to other serum biomarker in identifying patients with a periprosthetic shoulder infection [15]. In their study, a serum D-Dimer threshold of 0.6 mg/l showed a sensitivity and specificity of 61% and 74%, respectively.

In contrast to the results of Zmistowski et al. our study showed a good sensitivity but a poor specificity of D-Dimer in the diagnosis of shoulder PJI using a threshold of 0.75 mg/l. As mentioned above, the recommended threshold differs in recent studies and a universal threshold for D-Dimer in diagnosing PJI still remains unknown. Moreover, the inflammatory response of shoulder PJI may be much more different from that of hip and knee PJI due to the majority of low-virulence microorganisms, effecting the optimal serum D-Dimer threshold. In addition,, in our study, only two patients were infected with a high-virulent microorganism. As presented in recent studies, less virulent PJIs had been associated with low CRP values [6, 7, 9]. The hypothesis that the CRP response in patients with a PJI caused by low-virulent microorganisms may be week, may be also true for serum D-Dimer. Therefore, the detection of low-grade shoulder PJIs may be very challenging despite combination of both methods. However, combination of both methods may be used as a confirmatory test in the diagnosis of shoulder PJI meaning that the combination of an elevated D-Dimer and CRP serum level in an otherwise healthy patient with symptomatic shoulder arthroplasty indicates PJI with a high likelihood.

This study has some limitations. The small size of the study cohort can alter our results. However, the ROC analysis had a significance level of 0.026, which shows enough power of our sample size. While we don’t believe, that including more patients would change the results significantly, the conclusions drawn from this rather small cohort should be confirmed in future studies. Despite none of the patients in our cohort had a history of venous thrombosis or coagulation disorder including malignancy or autoimmune diseases, this was only based on the patient’s statements. The possibly raised D-Dimer levels in patients with venous thrombosis or coagulation disorder may alter our results and decrease the specificity of combination of CRP and D-Dimer. In these patients both methods may not be used as a confirmatory test.

## Conclusion

Serum D-Dimer showed a good sensitivity but a poor specificity for the diagnosis of shoulder PJI. Combination of D-Dimer and CRP led to improvement of the specificity, however, at the cost of sensitivity. Thus, combination of both methods may be used as a confirmatory test in the diagnosis of shoulder PJI but not to rule out infection.

## Data Availability

Not applicable.
